# A comparison of in-patient mental health rehabilitation services provided by the NHS and independent sector in England: patient and service characteristics

**DOI:** 10.1192/bjo.2026.12047

**Published:** 2026-07-24

**Authors:** Helen Killaspy, Christian Dalton-Locke, Mariam Adeleke, Nia Barber, Bella Branch, Caroline Sarah Clarke, Gerard Leavey, Artemis Igoumenou, Katherine Barrett, Megan Downey, Sophie Thomas, Jing Yi (Jessica) Weng, Rumana Z. Omar

**Affiliations:** Division of Psychiatry, University College London, UK; Mental Health Rehabilitation Services, North London NHS Foundation Trust, London, UK; Department of Statistical Science, University College London, UK; Research Department of Primary Care and Population Health, University College London, UK; Bamford Centre for Mental Health & Wellbeing, University of Ulster, UK; North London Service User Research Forum, Division of Psychiatry, University College London, UK

**Keywords:** In-patient treatment, mental health rehabilitation, NHS, independent sector, complex psychosis

## Abstract

**Background:**

In-patient mental health rehabilitation services provide specialist care to adults with complex psychosis. In England, there are more than 350 such services, half provided by the National Health Service (NHS) and half by the independent sector.

**Aims:**

To investigate whether NHS and independent sector in-patient mental health rehabilitation services differ.

**Method:**

We recruited a random sample of NHS and independent sector services across England. We collected cross-sectional data regarding service and patient characteristics through interviews with patients, clinicians and managers, and from healthcare record reviews.

**Results:**

We recruited 411 patients from 48 NHS services and 172 patients from 20 independent sector services. Service characteristics, including standardised quality scores, were similar. Most patients had a diagnosis of schizophrenia (NHS *n* = 237, 57.7%; independent sector *n* = 99, 57.6%) or schizoaffective disorder (NHS *n* = 88, 21.4%; independent sector *n* = 32, 18.6%). More than half had at least one comorbid condition, but more independent sector patients had at least two (NHS 131 of 411, 31.9%; independent sector 80 of 172, 46.5%; *P* = 0.001). Current admissions were twice as long for independent sector patients (NHS mean 1.2 years [s.d. 1.5]; independent sector mean 2.4 years [s.d. 2.8]). Standardised ratings of patients’ quality of life, autonomy, time use, satisfaction with care, social and everyday functioning, and challenging behaviours were similar. However, independent sector patients had more needs (NHS mean 6.5 [s.d. 3.2]; independent sector mean 7.9 [s.d. 3.9]; *P* < 0.001) and more had a history of fire setting (NHS 32, 7.9%; independent sector 28, 17.2%; *P* = 0.001).

**Conclusions:**

Both NHS and independent sector in-patient rehabilitation services support people with complex psychosis, but independent sector patients may have more complex conditions, which could explain their longer admissions.

Most people diagnosed with a severe mental illness such as schizophrenia recover, at least partially, but around a quarter will develop longer-term complex problems requiring specialist treatment from mental health rehabilitation services.^1,2^ These problems include persistent, severe ‘positive’ symptoms (hallucinations and delusions) and ‘negative’ symptoms (reduced motivation and emotional reactivity) of psychosis, as well as specific cognitive impairments affecting verbal fluency and organisational skills. A range of mental and physical health comorbidities may complicate recovery further; these may either pre-date the psychosis (such as intellectual impairment or developmental disorders) or develop alongside it (such as anxiety, depression, substance misuse, obesity, diabetes, and pulmonary and cardiovascular conditions). These problems often create difficulties for the individual in managing everyday tasks (such as self-care, housework, shopping, cooking and budgeting) and in their interpersonal skills, and they can lead to challenging behaviours.^3^ Many such individuals struggle to engage in community activities or gain employment, and more than half are vulnerable to self-neglect and/or sexual or financial exploitation.^4,5^ Owing to their complex needs, these patients tend to experience recurrent and often lengthy acute in-patient admissions, but our previous national research programmes have shown that when specialist mental health rehabilitation services are available, most people with complex psychosis progress well and graduate successfully from higher to lower levels of support over time, with associated cost benefits for the mental health and social care systems.^6,7^

Despite evidence of the effectiveness of mental health rehabilitation services, there has been major disinvestment in National Health Service (NHS)-provided in-patient mental health rehabilitation services across England, with increasing reliance on the independent sector to provide in-patient rehabilitation for NHS-funded patients. In 2018, the hospital inspectorate, the Care Quality Commission (CQC), surveyed all providers of in-patient mental health rehabilitation services in England.^8^ They estimated the cost of in-patient rehabilitation to be more than £500m annually, with the independent sector providing around half of the 3500+ beds in the country. They noted considerable regional variation in use of the independent sector and found that admissions to independent sector rehabilitation services were twice as long as those to NHS services, doubling the associated costs (the median stay in an independent sector unit was 444 days, compared with 230 days in the NHS). They also highlighted concerns about the social dislocation associated with being treated in the independent sector, as these services were usually much further from patients’ home areas than NHS services.

The CQC report prompted a national initiative by NHS England, launched in 2019, to encourage commissioners to invest in local NHS mental health rehabilitation services (Getting It Right First Time for Mental Health Rehabilitation).^9^ The National Institute for Health and Care Excellence (NICE) guideline on mental health rehabilitation for adults with complex psychosis (2020) included modelling of the potential cost savings if all in-patient rehabilitation were to be provided by the NHS; this was estimated at £52 000 per patient per year (a total of more than £100m per year).^10^ However, there have been no studies comparing the characteristics of patients, the quality of services or the outcomes associated with in-patient rehabilitation provided by the NHS and the independent sector. Previous small surveys have suggested that the NHS may be transferring patients with more complex needs to the independent sector, which could explain the longer admissions of these patients and mean that the potential cost savings from shifting to more local NHS provision may have been overestimated by NICE.^11,12^

The ACER (Assessing the Clinical and cost-Effectiveness of inpatient mental health Rehabilitation services provided by the NHS and independent sector) study aimed to compare NHS and independent sector in-patient mental health rehabilitation services in England in terms of: patient characteristics; service quality; patient, carer and staff experiences; clinical effectiveness; and cost-effectiveness. In this paper, we report findings for the first of five components of the ACER study, a national survey that addressed the following research questions:

- Do the sociodemographic characteristics, clinical characteristics, quality of life, and satisfaction with care of patients receiving in-patient mental health rehabilitation differ between the NHS and the independent sector?
- Does service quality differ between in-patient mental health rehabilitation units provided by the NHS and the independent sector?

Although the CQC report found that admissions were longer in the independent sector,^8^ and small surveys have suggested that this may be due to patients in the independent sector having more complex needs,^11,12^ the evidence for this has remained unclear. The present study was designed as a detailed survey of patient and service characteristics, and we adopted an exploratory approach rather than testing specific hypotheses.

## Method

### Scoping of eligible in-patient mental health rehabilitation units in England

We contacted all NHS mental health trusts and the four main independent sector providers of in-patient mental health rehabilitation in England (three of which agreed to participate) to identify services that were eligible for inclusion in the study. We confirmed details of the numbers and types of units provided, using the typology of in-patient rehabilitation services developed by the Royal College of Psychiatrists.^13^ We also recorded the number of beds per service and whether the service was single or mixed gender. Only three of the five types of rehabilitation service were eligible for this study (high-dependency, longer-term high-dependency and community rehabilitation units), as these are recommended components of a local mental health rehabilitation care pathway.^10^ The other two types are usually provided at a regional rather than local level and were not eligible for the study: these are specialist rehabilitation units for condition-specific groups (individuals with autism spectrum disorder, neurodegenerative disorder or brain injury) and forensic mental health rehabilitation units. Services with fewer than five beds were excluded to ensure adequate patient recruitment per service, aiming for an average eight participants per unit; this figure was based on recruitment in our previous national survey of NHS in-patient rehabilitation services.^6^

### Sampling of in-patient mental health rehabilitation units and participant sample size

We aimed to recruit a total of 500 patients, 250 from NHS and 250 from independent sector services. This target sample size was estimated to have sufficient power to detect a difference between the two sectors with respect to in-patient readmission rates at 18 months after recruitment (the primary outcome for component 3 of the ACER study). Further details of the sample size estimation are available in the ACER study protocol.^14^ On the basis of the mean number of beds at each of the sampled services and the recruitment and attrition rates from our previous study of NHS in-patient rehabilitation,^6^ we needed to recruit participants from 32 services in each sector to achieve our sample size. We randomly sampled 40 NHS and 40 independent sector services to account for the possibility of service closures over the course of the study. However, one of the three independent sector providers subsequently withdrew from the study, with the loss of 21 services from the sampling pool. Therefore, all 23 eligible services provided by the two remaining independent sector organisations, Priory Group and St Andrew’s Healthcare, were included, providing a smaller pool of patients for potential recruitment. We estimated that we could recruit 160 rather than 250 patients from these services. Consequently, a larger number of participants needed to be recruited from NHS services, and our recruitment ratio (NHS to independent sector) was therefore increased from 1:1 to 2.4:1. The target sample size in the NHS increased from 250 to 400, and the total target sample size increased from 500 to 560. Additional NHS services were randomly sampled and recruited to meet this new target.

### Patient recruitment

All patients of participating in-patient rehabilitation services were eligible for recruitment, except for those who were absent at the time of recruitment and those who lacked adequate English to provide informed consent. To avoid introducing sampling bias, we still recruited patients who were assessed as lacking the capacity to provide informed consent (see the ‘Consent’ section for the process regarding recruitment of patients lacking capacity). The researchers responsible for recruitment were trained by senior researchers with experience of recruiting people with complex psychosis to research studies. This training included the procedure for gaining informed consent and assessing a patient’s ability to retain important information about the study and to weigh the personal benefits and disadvantages of their participation. Recruitment was conducted from 8 June 2022 to 20 March 2024 with researchers spending up to 1 week in each unit.

### Data collection

The researchers who collected data were trained in how to complete the following standardised research interviews by senior researchers with experience of conducting research interviews with people with complex psychosis and mental healthcare staff.

#### Patient interviews

The following standardised measures were completed through face-to-face interviews with patient participants, taking around 30 min in total.

- The Manchester Short Assessment of Quality of Life^15^ assesses 11 aspects of daily life rated on a scale from 1 (couldn’t be worse) to 7 (couldn’t be better), generating a mean score between 1 and 7.
- The Resident Choice Scale^16^ measures autonomy. The patient rates the degree to which they have choice over 22 aspects of daily activities and the running of the in-patient unit on a four-point scale from 1 (I have no choice at all about this) to 4 (I have complete choice about this), providing a maximum possible score of 88, with higher scores indicating greater autonomy.
- The Time Use Diary^17^ assesses patients’ engagement in activities and can be completed by the patient or a staff member. Engagement and complexity of activities are assessed for the previous week during four periods each day (morning, lunchtime, afternoon and evening) and rated on a scale from 0 to 4 for each time period, giving a maximum possible score of 112, with higher scores denoting greater activity.
- The Client Assessment of Treatment^18^ measures satisfaction with care. The patient rates their satisfaction with seven aspects of their in-patient treatment on a scale from 0 (not at all satisfied) to 10 (totally satisfied), producing a mean score out of 10.
- The Recovering Quality of Life (ReQoL)^19^ ten-item version assesses quality of life in terms of personal recovery, with each item rated from 0 to 4, producing a total score between 0 and 40.
- The EuroQol 5-Dimension 5-Level questionnaire (EQ-5D-5L)^20^ is a five-item generic preference-based health-related quality-of-life measure, from which patients’ utility scores are calculated using standard methods.

For both the ReQoL – Utility Index^21^ and EQ-5D-5L utility scores,^22^ a score of 1 is the maximum and is indicates being in perfect health, and scores less than 1 indicate less than perfect health, with an anchor at zero (equivalent to being dead). Both the ReQoL and EQ-5D-5L were reported here for comprehensiveness but were primarily collected to provide data for a health economic analysis, which will be completed as part of a subsequent component of the ACER study.

#### Staff interviews

A staff member working in the in-patient rehabilitation service who knew the patient participant well was asked to complete the following assessments about them through a face-to-face interview with the researcher that took around 30 min.

- The Life Skills Profile^23^ assesses social and everyday functioning. It comprises 39 items, each rated on a four-point scale, with the most positive response scoring 4 and the least scoring 1, giving an overall score ranging from 39 to 156.
- The Special Problems Rating Scale (SPRS)^24^ assesses the presence and severity of 14 challenging behaviours on a scale of 0 (no problem) to 2 (frequent and/or extremely difficult to manage).
- The Clinical Alcohol and Drug Scale^25^ is a five-item scale used to rate, separately, alcohol and illicit substance use over the previous 6 months, with scores for each ranging from 1 indicating abstinence and 5 indicating dependence resulting in institutional admission. The degree of severity can also be summarised as a binary variable (problematic or non-problematic).
- The Camberwell Assessment of Needs Short Appraisal Scale^26^ assesses 22 domains of mental health and social need over the previous month as absent (0), met (1) or unmet (2). A need is rated as unmet even if the individual is receiving treatment and/or support for it, if it remains problematic. The scale generates score for total, met and unmet needs, and the proportion of met and unmet needs can be calculated.
- Time Use Diary^17^ (see patient interview measures).

#### Healthcare records

The researchers reviewed patient participants’ healthcare records to gather the following data:

- sociodemographic details (age, gender, ethnicity, civil status, highest educational attainment);
- primary and comorbid mental health diagnoses, and comorbid physical health conditions (categorised using the ICD-10);
- healthcare service use history (length of contact with mental health services, number of previous mental health admissions, start date of current in-patient admission, date of admission to current rehabilitation service, current Mental Health Act 1983 status);
- current (within the past 3 months) and previous recorded risks including: self-harm, suicide attempt(s), self-neglect, vulnerability to exploitation, aggression and/or violence to others (the severity of the most severe incident of aggression and/or violence were also noted, along with any previous admission(s) to a forensic mental health service);
- engagement in community-based activities over the past month (vocational, educational or leisure activities).

#### Unit manager

Data about the rehabilitation service were collected from the service manager through a face-to-face interview using the Quality Indicator for Rehabilitative Care (QuIRC),^27^ a standardised quality assessment tool for in-patient mental health rehabilitation services. It comprises 145 items covering the service setting (hospital or community) and size (number of beds); the average length of stay; the patient gender mix; the proportion detained under the Mental Health Act; patients’ degree of disability and/or need for staff assistance; staffing (full-time equivalents of different disciplines); staff training in rehabilitative skills; provision of staff supervision; staff turnover, vacancies and disciplinaries; provision of evidence-based pharmacological and psychosocial interventions, occupational therapy and facilitation of community activities (education, employment and leisure); interventions for physical healthcare and promotion; the therapeutic culture of the service; the degree to which patients are involved in developing their treatment and care plans; patient involvement in decisions about the running of the service; the protection of patients’ human rights; response to challenging behaviours, including use of de-escalation, restraint and seclusion; and the quality of the built environment. The QuIRC produces percentage ratings for seven domains of care (living environment, therapeutic environment, treatments and interventions, self-management and autonomy, social interface, human rights and recovery-based practice), with a higher percentage rating indicating better quality. It has excellent psychometric properties and takes around 1 h to complete.

### Data analysis

Patient and service characteristics are presented using descriptive statistics (frequencies and percentages for categorical variables; mean and standard deviations for continuous variables). We conducted statistical tests to compare a subset of variables chosen on the basis of clinical relevance and previous research suggesting that they may be associated with longer lengths of stay in in-patient rehabilitation services:^6^ primary diagnosis of personality disorder; having at least two comorbidities; length of contact with mental health services; being currently detained involuntarily; current challenging behaviour; current problematic substance use; total and unmet needs; current risk of self-harm, suicide or risk to others; previous admission to a forensic unit; and history of fire setting. Chi-squared and Fisher’s exact tests were used for categorical variables, and two-sample *t*-tests were used for continuous variables. To account for multiple comparisons, we applied Bonferroni correction by dividing the conventional alpha level (0.05) by the number of tests (14) to obtain an adjusted significance threshold of 0.003. All statistical tests were conducted using Stata version 19 for Windows (StataCorp, Texas, USA; https://www.stata.com/products/).

### Ethics

The authors assert that all procedures contributing to this work comply with the ethical standards of the relevant national and institutional committees on human experimentation and with the Helsinki Declaration of 1975, as revised in 2013. All procedures involving patients were approved by the NHS North East Newcastle and North Tyneside 2 Research Ethics Committee (ref. 22/NE/0067) on 8 June 2022. Approvals from the local NHS trusts and the two independent sector provider partners were also obtained before participant recruitment was started.

### Consent

Written information about the study was provided to potential participants, and these individuals were given time to consider whether to take part. The researchers answered any questions they had before obtaining written informed consent for participation. Patients were paid £20 for giving their time to participate in the research interview. For those who lacked capacity to make an informed decision with respect to their participation, the researcher asked a consultee (a relative or, if no relative were available, a staff member who knew the person well) whether they believed the person would like to participate or not. Consultees were provided with written information about the study and given the opportunity to ask the researcher questions. If the consultee believed the person would have agreed to participate if they had the capacity to do so, they were asked to complete and sign a consultee declaration form, and the researchers collected data as described but did not conduct a research interview with the patient.

## Results

### Unit characteristics

Twenty in-patient rehabilitation services provided by the two independent sector organisations were eligible by the time of recruitment (three having closed or changed their remit since scoping). All 20 were recruited, and a further 48 services were recruited from 22 NHS trusts. The characteristics of the participating services are shown in Table 1. Around one-third (6 of 20, 30%) of the independent sector services were categorised as longer-term high-dependency rehabilitation units, whereas this was the case for only 3 of 48 (6%) of the NHS units. Two-thirds (31 of 48, 65%) of NHS units and 2 of 20 (10%) independent sector units were mixed gender. One-third of units were in London or the south-east, with the rest spread across the country. The mean size of unit was similar in the 2 sectors (16 beds), with bed occupancy over 90% in both. The mean service quality (QuIRC domain ratings of units) was very similar in the two sectors.

**Table 1:** Characteristics of in-patient rehabilitation units by National Health Service and independent sector Table 1 long description.

Characteristic	National Health Service *N* = 48	Independent sector *N* = 20
Total number of beds	768	309
Mean (s.d.) beds per unit	15.9 (4.5)	15.6 (6.6)
Mean (s.d.) % bed occupancy	91.3 (16.3)	90.1 (13.1)
Unit type		
High-dependency rehabilitation unit, *n* (%)	28 (58)	9 (45)
Longer-term high-dependency rehabilitation unit, *n* (%)	3 (6)	6 (30)
Community rehabilitation unit, *n* (%)	17 (35)	5 (25)
Unit gender mix		
Male only, *n* (%)	15 (31)	13 (65)
Female only, *n* (%)	2 (4)	5 (25)
Mixed, *n* (%)	31 (65)	2 (10)
Unit location in England		
East, *n* (%)	2 (4)	4 (20)
London, *n* (%)	15 (31)	2 (10)
Midlands, *n* (%)	7 (15)	6 (30)
North-east, *n* (%)	7 (15)	3 (15)
North-west, *n* (%)	11 (23)	3 (15)
South-east, *n* (%)	3 (6)	2 (10)
South-west, *n* (%)	3 (6)	0 (0)
Service quality, QuIRC domain percentage scores		
Living environment, mean (s.d.)	72.5 (7.2)	73.5 (7.6)
Therapeutic eEnvironment, mean (s.d.)	69.3 (4.6)	68.6 (5.1)
Self-management and autonomy, mean (s.d.)	67.9 (7.3)	67.3 (6.8)
Social interface, mean (s.d.)	59.8 (10.3)	59.0 (9.3)
Human rights, mean (s.d.)	73.8 (7.2)	72.0 (7.7)
Treatments and interventions, mean (s.d.)	62.0 (6.4)	61.0 (5.9)
Recovery-based practice, mean (s.d.)	68.0 (6.3)	64.3 (6.6)

### Patient sociodemographic characteristics, diagnosis and mental health service use

Patient sociodemographic characteristics, diagnosis and mental health service use are shown in Table 2. Patient clinical status and engagement in community activities are shown in Table 3. The results of the statistical comparisons of the 14 preselected variables describing characteristics of NHS and independent sector patients are presented in Table 4.

**Table 2:** Patient sociodemographic characteristics, diagnoses and mental health service use

Characteristic	National Health Service *N* = 411	Independent sector *N* = 172
Male, *n* (%)	302 (73.5)	117 (68.0)
Mean (s.d.) age in years	42.3 (13.1)	42.6 (14.4)
Ethnicity, *n*	406	169
White, *n* (%)	277 (68.6)	119 (70.4)
Asian, *n* (%)	33 (8.2)	19 (11.2)
Black, *n* (%)	75 (18.6)	18 (10.7)
Mixed, *n* (%)	12 (3.0)	10 (5.9)
Other, *n* (%)	7 (1.7)	3 (1.8)
Highest level of educational qualification, *n*	196	63
Degree or higher, *n* (%)	22 (10.2)	6 (5.5)
Higher/further education but not to degree level, *n* (%)	12 (5.6)	2 (1.8)
A Level or equivalent, *n* (%)	30 (14.0)	18 (16.5)
GCSE or equivalent, *n* (%)	55 (25.6)	29 (26.6)
Other qualifications, *n* (%)	14 (6.5)	5 (4.6)
No qualifications, *n* (%)	82 (38.1)	49 (45.0)
Civil status, *n*	387	161
Single and never married or in a civil partnership, *n* (%)	326 (84.2)	137 (85.1)
Married or civil partnership, *n* (%)	15 (3.9)	6 (3.7)
Divorced or separated, *n* (%)	40 (10.3)	15 (9.3)
Widowed, *n* (%)	6 (1.6)	3 (1.9)
Primary mental health diagnosis (ICD-10 code), *n*	411	172
Schizophrenia (F20–F24 and F26–F29), *n* (%)	237 (57.7)	99 (57.6)
Schizoaffective disorder (F25), *n* (%)	88 (21.4)	32 (18.6)
Bipolar affective disorder (F30–F31), *n* (%)	27 (6.6)	9 (5.2)
Personality disorder (F60–F69), *n* (%)	20 (4.9)	23 (13.4)
Depression or anxiety (F32–F48), *n* (%)	14 (3.4)	1 (0.6)
Substance misuse (F10–F19), *n* (%)	6 (1.5)	1 (0.6)
Developmental disorder (F80–F89), *n* (%)	5 (1.2)	0 (0.0)
Organic disorder (F00–F09), *n* (%)	1 (0.2)	4 (2.3)
Adult attention-deficit hyperactivity disorder (F90), *n* (%)	1 (0.2)	0 (0.0)
Other mental health disorder, *n* (%)	12 (2.9)	3 (1.7)
Any mental health comorbid condition, *n* (%)	209 (50.9)	98 (57.0)
Substance misuse (F10–F19), *n* (%)	114 (54.5)	44 (44.9)
Depression or anxiety (F32–F48), *n* (%)	36 (17.2)	20 (20.4)
Personality disorder (F60–F69), *n* (%)	25 (12.0)	22 (22.4)
Intellectual disability (F70–F79), *n* (%)	13 (6.2)	12 (12.2)
Any physical health comorbid condition, *n* (%)	145 (35.3)	83 (48.3)
Endocrine or metabolic disease (E00–E99), *n* (%)	59 (41.6)	43 (54.4)
Disease of the circulatory system (I00–I99), *n* (%)	44 (31.0)	18 (22.8)
Disease of the respiratory system (J00–J99), *n* (%)	31 (21.8)	21 (26.6)
Any mental or physical health comorbidity, *n* (%)	281 (68.4)	134 (77.9)
Length of contact with mental health services (years), *n*	411	170
Mean (s.d.)	16.3 (11.2)	20.4 (12.5)
Mean (s.d.) number of previous mental health admissions	5.1 (4.6)	5.4 (5.0)
Current Mental Health Act (1983) status, *n*	411	172
Voluntary, *n* (%)	110 (26.8)	11 (6.4)
Section 2, *n* (%)	2 (0.5)	0 (0.0)
Section 3, *n* (%)	241 (58.6)	113 (65.7)
Section 37, *n* (%)	7 (1.7)	3 (1.7)
Section 37/41, *n* (%)	44 (10.7)	34 (19.8)
Other section, *n* (%)	7 (1.7)	11 (6.4)
Mean (s.d.) total length in years of current admission	3.0 (4.9)	6.8 (8.4)
Mean (s.d.) years in current in-patient rehabilitation unit	1.2 (1.5)	2.4 (2.8)

**Table 3:** Patient clinical status and engagement in activities Table 3 long description.

Characteristic	National Health Service *N* = 411	Independent sector *N* = 172
Quality of life (MANSA) score^a^, *n*	383	164
Mean (s.d.)	4.8 (1.0)	4.9 (1.0)
Recovery quality of life (ReQoL)^a^, *n*	386	165
Mean (s.d.)	25.6 (8.2)	26.0 (7.7)
Recovery quality of life (ReQoL)^a^ utility score, *n*	387	165
Mean (s.d.)	0.81 (0.21)	0.82 (0.21)
EQ-5D-5L utility score^a^, *n*	386	165
Mean (s.d.)	0.76 (0.26)	0.75 (0.26)
Autonomy (RCS) score^a^, *n*	322	156
Mean (s.d.)	62.4 (9.0)	62.5 (9.4)
Patient-rated time use (TUD)^a^, *n*	354	156
Mean (s.d.)	42.2 (10.7)	39.5 (10.3)
Patient satisfaction with care (CAT)^a^, *n*	377	164
Mean (s.d.)	7.0 (2.3)	7.1 (2.4)
Social and everyday function (LSP)^a^, *n*	376	160
Mean (s.d.)	128.9 (14.8)	130.2 (15.3)
Challenging behaviours (SPRS)^b^, *n*	411	172
Any challenging behaviour, *n* (%)	374 (91.0)	169 (98.3)
Total SPRS score^b^, *n*	388	154
Mean (s.d.)	1.9 (2.2)	2.0 (2.6)
Substance misuse (CADS)^a^, *n*	410	172
Problematic alcohol use, *n* (%)	38 (9.3)	7 (4.1)
Mean (s.d.) alcohol use score	1.4 (0.7)	1.2 (0.5)
Problematic use of illicit substances, *n* (%)	52 (12.7)	11 (6.4)
Mean (s.d.) illicit substance use score	1.4 (0.8)	1.2 (0.6)
Staff-rated time use (TUD)^a^, *n*	387	159
Mean (s.d.)	39.4 (10.4)	38.7 (11.0)
Staff-rated total needs (CANSAS)^b^, *n*	411	172
Mean (s.d.)	6.5 (3.2)	7.9 (3.9)
Proportion of needs that are unmet (CANSAS)^b^, *n*	411	172
Mean (s.d.)	0.4 (0.3)	0.4 (0.3)
Current risk of self-harm or suicide, *n*	411	172
Yes, *n* (%)	30 (7.3)	23 (13.4)
Previous history of self-harm, *n*	407	169
Yes, *n* (%)	138 (33.9)	85 (50.3)
Previous risk of suicide, *n*	406	168
Yes, *n* (%)	166 (40.9)	91 (54.2)
Current risk of self-neglect, *n*	411	171
Yes, *n* (%)	175 (42.6)	68 (39.8)
Previous risk of self-neglect, *n*	407	168
Yes, *n* (%)	324 (79.6)	131 (78.0)
Current vulnerability to sexual and/or financial exploitation, *n*	409	172
Yes, *n* (%)	198 (48.4)	88 (51.2)
Previous vulnerability to sexual and/or financial exploitation, *n*	409	170
Yes, *n* (%)	280 (68.5)	128 (75.3)
Current risk to others, *n*	411	171
Yes, *n* (%)	45 (10.9)	25 (14.6)
Previous risk to others, *n*	406	170
Yes, *n* (%)	266 (65.5)	139 (81.8)
Most severe incident of aggression/violence to other, *n*	398	165
Homicide, *n* (%)	6 (1.5)	12 (7.3)
Bodily harm requiring medical attention, *n* (%)	95 (23.9)	60 (36.4)
Bodily harm not requiring medical attention, *n* (%)	120 (30.2)	56 (33.9)
Verbal abuse and/or threats, *n* (%)	93 (23.4)	28 (17.0)
None, *n* (%)	84 (21.1)	9 (5.5)
Previously admitted to a forensic mental health unit, *n*	398	162
Yes, *n* (%)	96 (24.1)	57 (35.2)
History of fire setting, *n*	405	163
Yes, *n* (%)	32 (7.9)	28 (17.2)
Engaging in vocational rehabilitation, *n*	409	172
Open and/or competitive employment, *n* (%)	3 (0.7)	0 (0.0)
Voluntary, protected and/or sheltered employment, *n* (%)	10 (2.4)	18 (10.5)
None, *n* (%)	396 (96.8)	154 (89.5)
Engaging in education, *n*	411	171
University or college course, *n* (%)	6 (1.5)	4 (2.3)
Recovery College or similar, *n* (%)	17 (4.1)	13 (7.6)
None, *n* (%)	388 (94.4)	154 (90.1)
Participated in exercise, sport or gym in the past month, *n*	407	171
Yes, *n* (%)	141 (34.6)	69 (40.4)
Went out to a café/restaurant in the past month, *n*	398	167
Yes, *n* (%)	286 (71.9)	119 (71.3)
Went to the cinema in the past month, *n*	401	169
Yes, *n* (%)	59 (14.7)	39 (23.1)
Went shopping in the past month, *n*	410	172
Yes, *n* (%)	374 (91.2)	151 (87.8)
Went to a place of worship in the past month, *n*	404	169
Yes, *n* (%)	40 (9.9)	18 (10.7)
Went to a park or other green space in the past month, *n*	390	167
Yes, *n* (%)	243 (62.3)	113 (67.7)
Has been outside the unit/service campus in the past month, *n*	411	172
Yes, *n* (%)	394 (95.9)	156 (90.7)

a. Higher score indicates better outcome: MANSA (Manchester Short Assessment of Quality of Life) scores range from 1 to 7; ReQoL (Recovering Quality of Life) scores range from 0 to 40; ReQoL and EQ-5D-5L utility scores range form 0 to 1; RCS (Resident Choice Scale) scores range from 22 to 88; LSP (Life Skills Profile) score ranges from 39 to 156; CADS (Clinician Alcohol and Drug Scale) score ranges from 1 to 5; TUD (Time Use Diary) score ranges from 0 to 112; CAT (Client Assessment of Treatment) scores range from 0 to 10.

b. Higher score indicates worse outcome: SPRS (Special Problems Rating Scale) score ranges from 0 to 32; CANSAS (Camberwell Assessment of Needs Short Appraisal Scale) score ranges from 0 to 22.

**Table 4:** Comparison of selected patient characteristics by sector Table 4 long description.

Characteristic	National Health Service (*n* = 411)	Independent sector (*n* = 172)	*P*-value^a^
Primary mental health diagnosis of personality disorder (F60–69), *n* (%)	20 (4.9)	23 (13.4)	0.002*
Comorbidity of any mental or physical health condition, *n* (%)	281 (68.4)	134 (77.9)	0.02**
Two or more comorbid mental or physical health conditions, *n* (%)	131 (31.9)	80 (46.5)	0.001**
Mean (s.d.) years of contact with mental health services	16.3 (11.2)	20.4 (12.5)	<0.001***
Currently detained involuntarily, *n* (%)	301 (73.2)	161 (93.6)	<0.001*
Any challenging behaviour (SPRS), *n* (%)	374 (91.0)	169 (98.3)	0.001*
Problematic alcohol use (CADS), *n* (%)	38 (9.3)	7 (4.1)	0.04*
Problematic drug use (CADS), *n* (%)	52 (12.7)	11 (6.4)	0.03**
Total needs (CANSAS), mean (s.d.)	6.5 (3.2)	7.9 (3.9)	<0.001***
Unmet needs (CANSAS), mean (s.d.)	0.4 (0.3)	0.4 (0.3)	0.10***
Current risk of self-harm or suicide, *n* (%)	30 (7.3)	23 (13.4)	0.02**
Current risk to others, *n* (%)	45 (10.9)	25 (14.6)	0.21**
Previously admitted to a forensic unit, *n* (%)	96 (24.1)	57 (35.2)	0.008**
History of fire setting, *n* (%)	32 (7.9)	28 (17.2)	0.001**

CADS, Clinician Alcohol and Drug Scale; CANSAS, Camberwell Assessment of Needs Short Appraisal Scale; SPRS, Special Problems Rating Scale.

a. We conducted 14 comparisons and applied a Bonferroni correction, resulting in an adjusted significance level of 0.05/14 = 0.003. Unadjusted *P*-values are reported; findings were considered statistically significant if *P* < 0.003 (shown in bold).

*Fisher’s exact test; **chi-squared test; ***two-sample *t*-test.

A total of 411 of 672 (61%) eligible patients were recruited from NHS services, of whom 22 (5% of those recruited) lacked capacity. From the independent sector services, 172 of 261 (66%) eligible patients were recruited, of whom seven (4% of those recruited) lacked capacity. In both the NHS and independent sector, more than two-thirds were male and White, with a mean age of 42 years. More than 80% were single and had never been married or in a civil partnership, and a relatively large percentage (NHS: 38%, independent sector: 45%) had not achieved any educational qualifications (although there were considerable missing data for this variable). The vast majority had a primary diagnosis of schizophrenia (NHS: *n* = 237, 57.7%; independent sector: *n* = 99, 57.6%), schizoaffective disorder (NHS: *n* = 88, 21.4%; independent sector: *n* = 32, 18.6%) or bipolar affective disorder (NHS: *n* = 27, 6.6%; independent sector: *n* = 9, 5.2%). More patients of independent sector than NHS services had a primary diagnosis of personality disorder, although the numbers with this primary diagnosis were very small (NHS: 20 of 411 [4.9%], independent sector: 23 of 172 [13.4%]; *P* = 0.002). More than half of the patients in both sectors had at least one comorbid mental health condition (NHS: 209 of 411, 50.9%; independent sector: 98 of 172, 57.0%), and more than one-third of NHS (145 of 411, 35.3%) and almost half of independent sector patients (83 of 172, 48.3%) had at least one physical health comorbidity. More patients of independent sector than NHS services had at least two comorbid conditions (NHS: 131 of 411 [31.9%]; independent sector: 80 of 172 [46.5%]; *P* = 0.001). Patients in both sectors had been known to mental health services for many years, but those in the independent sector had longer histories of contact (NHS: mean 16.3 years [s.d. 11.2], independent sector: mean 20.4 years [s.d. 12.5]; *P* < 0.001). Patients in both sectors had experienced a mean of five previous admissions. Although most were currently detained involuntarily, this was more commonly the case for those treated in the independent sector (NHS: 301 of 411 [73.2%], independent sector: 161 of 172 [93.6%]; *P* < 0.001). The mean length of stay in the rehabilitation unit for independent sector patients was twice that of NHS patients (NHS: 1.2 years [s.d. 1.5], independent sector: 2.4 years [s.d. 2.8]), and their total mean admission length, including the period before transfer to the rehabilitation unit, was more than double that of NHS patients (NHS: 3.0 years [s.d. 4.9], independent sector: 6.8 years [s.d. 8.4]).

### Patient clinical status and engagement in activities

Table 3 shows patients’ clinical status and engagement in activities. Using our threshold for statistical significance, we found no differences between the two sectors in standardised ratings of patients’ quality of life (Manchester Short Assessment of Quality of Life),^15^ generic health-related quality of life (utility scores) using either the ReQoL – Utility index^21^ or the EQ-5D-5L,^22^ autonomy (Resident Choice Scale),^16^ time use (Time Use Diary),^17^ satisfaction with care (Client Assessment of Treatment),^18^ social and everyday functioning (Life Skills Profile)^23^ and challenging behaviours (SPRS).^24^ However, although most patients were rated as having at least one current challenging behaviour (SPRS),^24^ this was the case for slightly more of the independent sector patients (NHS: 374 of 411 [91.0%], independent sector: 169 of 172 [98.3%]; *P* = 0.001). Although we found no statistically significant difference between the two sectors in the percentage of patients rated as having problematic alcohol or illicit substance use (Clinical Alcohol and Drug Scale),^25^ independent sector patients had more needs as assessed by the Camberwell Assessment of Needs Short Appraisal Scale^26^ (NHS: total mean 6.5 [s.d. 3.2], independent sector: total mean 7.9 [s.d. 3.9]; *P* < 0.001) and included a higher percentage with a history of fire setting (NHS: 32 [7.9%], independent sector: 28 [17.2%]; *p* = 0.001). With regard to current risks, there were no statistically significant differences between the two sectors with respect to the percentage of patients considered to be at risk of sexual or financial vulnerability, self-neglect or self-harm, or the percentage considered to pose a risk to others. The most commonly reported current risks were sexual and/or financial vulnerability (NHS: 198 [48.4%], independent sector: 88 [51.2%]) and self-neglect (NHS: 175 [42.6%], independent sector: 68 [39.8%]).

Very few patients in either sector were engaging in vocational rehabilitation or educational activities, but most were engaging in at least one leisure activity (most commonly shopping or going to a café), and more than 90% in both sectors had been outside the unit or hospital campus in the past month (NHS: 394 of 411 [95.9%], independent sector: 156 of 172 [90.7%]). More than one-third were participating in regular exercise in both sectors (NHS: 141 of 407 [34.6%], independent sector: 69 of 171 [40.4%]).

## Discussion

This is the first national study to describe in-patient mental health rehabilitation services provided by the NHS and independent sector in England and the characteristics of their patients. We planned to randomly sample services in both sectors, and stratify by service type, gender mix and region. However, owing to the withdrawal of one independent sector provider, there was a reduced sampling pool; this meant we could not randomly select the independent sector services and included all eligible services instead. Consequently, there were differences between the two sectors in some service characteristics which may at least partially explain some of the observed differences in patient characteristics. Importantly, a higher proportion of the sampled NHS services were community rehabilitation units compared with the independent sector services, and a higher proportion of the independent sector services were longer-term high-dependency rehabilitation units compared with the NHS. These different types of service may be associated with differences in the clinical characteristics of patients, reflecting their progress in the process of rehabilitation; for example, if a local rehabilitation care pathway includes different types of unit, patients are likely to transition from the high-dependency unit to the community rehabilitation unit as their condition stabilises. There is, however, considerable overlap between these service types. Many local rehabilitation services include only one type of rehabilitation unit, and the length of stay in some ‘longer-term’ high-dependency rehabilitation units is shorter than that in some high-dependency rehabilitation units; moreover, some community rehabilitation units take detained patients, whereas others do not. We therefore included all three types of unit in this study to ensure comprehensive coverage of all types of ‘standard’ (non-specialist) in-patient rehabilitation in England. The services recruited were of similar size in terms of bed numbers and were rated similarly with respect to all domains of quality.

There were also many similarities in patient characteristics between the two sectors, with more than two-thirds of patients being male and White, and a mean age of 42 years. The vast majority had a primary diagnosis of schizophrenia, schizoaffective disorder or bipolar affective disorder and had been known to mental health services for many years, with numerous (five) previous admissions. More than two-thirds had at least one comorbid condition, and more than 90% presented with ongoing challenging behaviour(s). Around a quarter had been previously admitted to a forensic mental health unit. The data suggest that mental health rehabilitation services in both sectors are continuing to focus on providing specialist input for people with complex psychosis, in keeping with NICE guidance.^10^ We found no statistically significant differences between patients in the two sectors with respect to our standardised ratings of quality of life, autonomy, satisfaction with care, time use, social and everyday functioning, and substance misuse.

We found that the average length of admission in the independent sector services was more than twice that of NHS services. This finding concurred with the CQC’s 2018 report, in which the median length of stay in in-patient mental health rehabilitation services was noted as 1.3 years in NHS units and 2.6 years in independent sector units.^8^ Our data also suggest that the length of stay in both sectors has increased since the CQC survey, but more so in the independent sector; we found the median length of stay in the NHS services to be 1.4 years (interquartile range: 0.7 to 3.1), compared with 3.4 years (interquartile range: 1.6 to 8.5) in the independent sector services.

Our statistical comparisons of selected patient characteristics identified some differences between the two sectors that may at least partially explain the discrepancy in length of stay. However, we stress the exploratory nature of these comparisons, given that the study was not specifically powered to investigate these differences (see the ‘Limitations’ section). Although mental and physical health comorbidities were common for patients in both sectors, a higher percentage of those being treated in the independent sector than those treated in the NHS had at least two comorbid conditions. Furthermore, although the vast majority of patients of both sectors presented with current challenging behaviours, this was more common for those being treated in the independent sector (although there was no difference in ratings of severity of these behaviours between sectors). Patients of independent sector services were also rated as having more needs than NHS patients and were more likely to have a history of fire setting. Taken together, these findings indicate that independent sector rehabilitation services may be working with patients with a more complex profile than the NHS. This is further supported by our finding that although most patients in both sectors were detained involuntarily, this was the case for more of those treated in the independent sector.

In our previous national study of NHS in-patient mental health rehabilitation services,^6^ we found that a history of fire setting was associated with being less likely to be discharged to the community (owing to problems identifying supported accommodation services willing to accept individuals with this type of risk history). Other studies of in-patient mental health rehabilitation services have also found that patients with more challenging behaviour were less likely to achieve successful community discharge.^28–30^ It is therefore plausible that the longer length of stay in independent sector units could be because these units have patients with more of these types of characteristic. However, our previous study also found length of admission in the rehabilitation unit to be negatively associated with successful community discharge,^6^ a finding corroborated by other authors.^28^ This could be because those who are more difficult to treat require longer to recover adequately to be ready for discharge, but it is also possible that longer stays are detrimental owing to deskilling, and thus time itself becomes an impediment to discharge. However, it is reassuring that services in both sectors were of similar quality and, importantly, did not differ in provision of recovery-based practice (a collaborative, enabling approach), which has been shown to be associated with successful discharge from in-patient rehabilitation services.^6^ Our finding that most patients in both sectors were engaged in leisure activities also suggests that services in both sectors are providing a proactive rehabilitative approach rather than understimulating environments, refuting CQC criticism that the quality of care provided by independent sector rehabilitation units is inferior to that provided by the NHS.^8^ The low percentage of patients engaged in other activities, including educational and work-related activities, is likely to reflect the level of disability of individuals in this group and the complex nature of their conditions, although it could also reflect a lack of availability of such activities.

Of note, although relatively few patients had a primary diagnosis of personality disorder, the percentage with this diagnosis was higher in the independent sector than the NHS services, and this difference was statistically significant. Further research is required to confirm that this difference generalises to other independent sector rehabilitation services compared with the NHS. This is important given recent publication of commissioning guidance for mental health rehabilitation services that encourages services to broaden their remit to diagnoses other than complex psychosis, despite there being no evidence to support this being safe or effective.^31^ Mental health rehabilitation is a specialism that focuses on people with complex psychosis, and services may therefore struggle to work successfully with people with a primary diagnosis of personality disorder, potentially leading to longer lengths of stay or subsequent transfers to other in-patient services.

Concerns about the NHS ‘exporting’ its most complex patients to the independent sector have been repeatedly raised over many years by the Royal College of Psychiatrists, the media and other organisations.^32–35^ The latest report from NHS England’s Getting It Right First Time for Mental Health Rehabilitation initiative suggests that the independent sector still provides at least half of the 3500 or so in-patient mental health rehabilitation beds in England, but with huge variation in this across the country, from 0 to 26 beds per 100 000 weighted regional population.^36^ This suggests that NHS services in at least some areas can manage even the most complex patients, whereas others outsource their in-patient rehabilitation to the independent sector. As independent sector services tend to be further from patients’ homes than NHS services, this variability represents a ‘national lottery’ by postcode in terms of access to appropriate treatment as close to home as possible. Indeed, the social dislocation associated with being treated a long way from home has also been suggested as a possible explanation for the longer lengths of stay in independent sector services, as it makes it difficult for the patients’ family and their local community team to remain closely involved, potentially impeding discharge planning.^34^ Our data cannot explain why length of stay, particularly in the independent sector, seems to have increased since 2018, but one possibility is that the ongoing focus on restructuring local NHS community mental health services since publication of NHS England’s Community Mental Health Framework in 2019,^37^ may have had a negative impact on links between independent sector services and local community teams that enable patients’ discharge. The framework does not mention community teams’ responsibilities towards people placed in in-patient settings outside their local area. The NICE guideline^10^ provides clear recommendations on this, but these do not appear to be implemented consistently. The present study highlights the complexities that need to be considered when comparing patient outcomes across the NHS and independent sector with respect to in-patient mental health rehabilitation. Our findings may help to inform future policy and commissioning of in-patient rehabilitation services, but they do not provide definitive evidence. Further components of the ACER study will report on the clinical and cost-effectiveness of the two sectors, taking into account the differences in service and patient characteristics identified in this study.

### Limitations

This study provides cross-sectional descriptive data on in-patient mental health rehabilitation services and patient characteristics provided by the NHS and independent sector in England. The different proportions of rehabilitation service type in each sector recruited to this study may explain, at least partially, some of the differences we identified. We acknowledge that our sample size was based on that required to compare the effectiveness of the two sectors in the separate, prospective component of the ACER study; therefore, the statistical comparisons of patient and service characteristics presented here are exploratory and should be interpreted with caution. Nevertheless, the differences in service and patient characteristics we identified will be taken into account in the subsequent clinical and cost-effectiveness components of the ACER study. Further, although this component of the study could only include two of the four main independent sector providers of in-patient rehabilitation, limiting generalisability, a separate component of the ACER study will report on the effectiveness of the two sectors using a comprehensive national data-set, which will produce more generalisable findings. An additional qualitative component of the ACER study will also report on staff and patient experiences and perspectives on these services.

## Data Availability

The data that support the findings of this study are available from the corresponding author, H.K., upon reasonable request.

## Table 1 Long description

The table compares characteristics of in-patient rehabilitation units by National Health Service and independent sector. It has 15 rows and 4 columns. The columns are Total number of beds, Mean (s.d.) beds per unit, Mean (s.d.) % bed occupancy, Unit type, n (%), Unit gender mix, n (%), Unit location in England, n (%), and Service quality, mean (s.d.) QuIRC domain percentage scores. The rows are labeled with different characteristics and their respective values for National Health Service and independent sector. Row 1: Total number of beds, 768, 309. Row 2: Mean (s.d.) beds per unit, 15.9 (4.5), 15.6 (6.0). Row 3: Mean (s.d.) % bed occupancy, 91.3 (16.3), 90.1 (13.1). Row 4: High-dependency rehabilitation unit, 28 (58%), 9 (45%). Row 5: Longer-term high-dependency rehabilitation unit, 3 (6%), 6 (30%). Row 6: Community rehabilitation unit, 17 (35%), 5 (25%). Row 7: Male only, 15 (31%), 13 (65%). Row 8: Female only, 2 (4%), 5 (25%). Row 9: Mixed, 31 (65%), 2 (10%). Row 10: East, 2 (4%), 4 (20%). Row 11: London, 15 (31%), 2 (10%). Row 12: Midlands, 7 (15%), 6 (30%). Row 13: North-east, 7 (15%), 3 (15%). Row 14: North-west, 11 (23%), 3 (15%). Row 15: South-east, 3 (6%), 2 (10%). Row 16: South-west, 3 (6%), 0 (0%). Row 17: Living environment, 72.5 (7.2), 73.5 (7.6). Row 18: Therapeutic environment, 69.3 (4.6), 68.6 (5.1). Row 19: Self-management and autonomy, 67.9 (7.3), 67.3 (6.8). Row 20: Social interface, 59.8 (10.3), 59.0 (9.3). Row 21: Human rights, 73.8 (7.2), 72.0 (7.7). Row 22: Treatments and interventions, 62.0 (6.4), 61.0 (6.9). Row 23: Recovery-based practice, 68.0 (6.3), 64.3 (6.0).

Navigate back to Table 1.

## Table 3 Long description

The table compares patient clinical status and engagement in community activities between the National Health Service (N=411) and the Independent sector (N=172). It includes various metrics such as quality of life, recovery quality, utility scores, autonomy, patient-rated time use, patient satisfaction with care, social and everyday function, challenging behaviors, substance misuse, risk of self-harm or suicide, vulnerability to exploitation, risk to others, history of aggression or violence, admission to forensic mental health units, engagement in vocational rehabilitation, employment status, education, physical exercise, social activities, and religious activities. Each row provides specific data points for both sectors, highlighting differences and similarities in patient outcomes and engagement levels.

Navigate back to Table 3.

## Table 4 Long description

The table compares selected patient characteristics by sector, including diagnosis, mental health service use, and clinical status. It has 17 rows and 5 columns. The columns are labeled National Health Service (n = 411), Independent sector (n = 172), and P-value. The rows are labeled with various patient characteristics and their corresponding values. Row 1: Primary mental health diagnosis of personality disorder (F60-69), 20 (4.9 percent), 23 (13.4 percent), 0.002. Row 2: Comorbidity of any mental or physical health condition, 281 (68.4 percent), 134 (77.9 percent), 0.02. Row 3: Two or more comorbid mental or physical health conditions, 131 (31.9 percent), 80 (46.5 percent), 0.001. Row 4: Mean (s.d.) years of contact with mental health services, 16.3 (11.2), 20.4 (12.5), <0.001. Row 5: Currently detained involuntarily, 301 (73.2 percent), 161 (93.6 percent), <0.001. Row 6: Any challenging behaviour (SPRS), 374 (91.0 percent), 169 (98.3 percent), 0.001. Row 7: Problematic alcohol use (CADS), 38 (9.3 percent), 7 (4.1 percent), 0.04. Row 8: Problematic drug use (CADS), 52 (12.7 percent), 11 (6.4 percent), 0.03. Row 9: Mean (s.d.) total needs (CANSAS), 6.5 (3.2), 7.9 (3.9),<0.001. Row 10: Mean (s.d.) unmet needs (CANSAS), 0.4 (0.3), 0.4 (0.3), 0.10. Row 11: Current risk of self-harm or suicide, 30 (7.3 percent), 23 (13.4 percent), 0.02. Row 12: Current risk to others, 45 (10.9 percent), 25 (14.6 percent), 0.21. Row 13: Previously admitted to a forensic unit, 96 (24.1 percent), 57 (35.2 percent), 0.008. Row 14: History of fire setting, 32 (7.9 percent), 28 (17.2 percent), 0.001.

Navigate back to Table 4.
